# A cross-sectional survey and follow up study on major dairy health problems in large and small scale urban farms in Mekelle, Tigray, Ethiopia

**DOI:** 10.1186/s13104-018-3347-0

**Published:** 2018-04-10

**Authors:** Mebrahtu Tedla, Feven Mehari, Hassen Kebede

**Affiliations:** 1 Department of Biomedical Sciences, College of Veterinary Medicine and Animal Sciences, University of Gondar, P.O.box:196, Gondar, Ethiopia; 2 Department of Clinical Studies, College of Veterinary Medicine and Animal Sciences, University of Gondar, P.O.box:196, Gondar, Ethiopia

**Keywords:** Dairy farm, Infectious disease, Reproductive problems, Parasitism, Cross-sectional survey, Follow up, Mekelle

## Abstract

**Objective:**

This study was conducted with the objective of estimating the incidence of major dairy health problems in the area.

**Result:**

From a cross-sectional survey (n = 475) and follow up study (n = 68), an overall incidence of 43.00 and 29.02% was reported respectively. This study showed biting fly (9.51%), respiratory problems (7.80%), mastitis (5.13%), actinomycosis (5.12%), dystocia (4.42%), endoparasites (3.81%), retention fetal membrane (3.63%), tick infestation (2.91%), lameness (2.94%), vaginal and uterine prolepses (2.51%), skin related problem (1.70%) and abortion (1.70%) were the main dairy health problems identified. In addition, the follow up study revealed; retention fetal membrane (5.91%), tick infestation (5.91%), respiratory problem (2.91%), mastitis (2.94%), endoparasites (2.94%), lameness (2.94%), dystocia (2.94%), actinomycosis (1.53%) and skin related problems (1.53%). The incidence of dairy reproductive problems showed statistically significant variation among local and cross breeds (P < 0.05). Incidence of infectious diseases among dairy cows managed under intensive and semi-intensive management systems showed a significant difference (P < 0.05). Moreover, incidence of physical injury was also showed a significant difference among animal breeds and management system (P < 0.05). However, reproductive problems among management system and infectious diseases among breeds showed a significant difference (P > 0.05). Overall, this study showed dairy animals are exposed to various type of diseases.

## Introduction

Livestock population represents the major resource and it forms an integral part of the agricultural production system in Ethiopia [1]. However, the production and productivity of livestock is still marginalized and the most contributing factors are the presence of widely distributed animal diseases which caused loss of meat and milk, and reduction in animal traction power as a result of disease morbidity [2]. Parasitic disease caused by helminths, protozoa and arthropods causes serious economic losses than disease caused by bacteria and viruses. However, the impact of animal disease to livestock owners is less understood [3]. It is known that the low livestock productivity in the tropics is attributed to poor genetic potential, malnutrition, inadequate management practices. This mainly due to most of the cattle are located in rural areas where less attention is given to animals in terms of care and treatment when high incidence of disease burden prevails which is the principal cause of morbidity and mortality [4]. There is a good progress in reducing the incidences of economically important livestock disease through selection, crossbreeding and establishment of intensive management systems. In addition, the opportunities for development of these programs are greater in dairy industry because of the need to monitor production and health on daily basis [5].

Previously, clinical studies on major livestock disease has been reported in other parts of the region and findings showed that different infectious diseases such as actinomycosis (16.00%), mastitis (15.00%), tick infestation (10.00%), respiratory diseases (9.16%), gastro intestinal parasitism (9.16%), and black leg (6.00%) in cattle, and pasteurellosis (38, 31%), contagious ecthyma (12, 10.00%), tick infestation (9.00, 0.00%), mite infestation (9.00, 10.00%), sheep and goat pox (9.00, 10.00%) in sheep and goat respectively were the most common diseases identified in different regions [6]. In addition, a higher prevalence (43.50%) of gastrointestinal parasites in small ruminants were reported in southern part of the country [7]. The major biological and socio- economical factors attributing to the low productivity includes the low genetic potential and performance, poor nutrition, and traditional ways of husbandry system [3].

Like any other developmental efforts occurring in the region, the regional bureau of agriculture and rural development has designed strategic plan to improve livestock productivity and efforts has been made to improve the genetic potential of dairy cows through selective breeding. However, no comprehensive study has been done to address the overall health constraints of dairy cows in this area.

Therefore, this study was conducted to assess dairy cattle for the presence of health problems that could have a significant economic impact to the private farm operators.

## Main text

### Methods

#### Study area

**Fig. 1:**
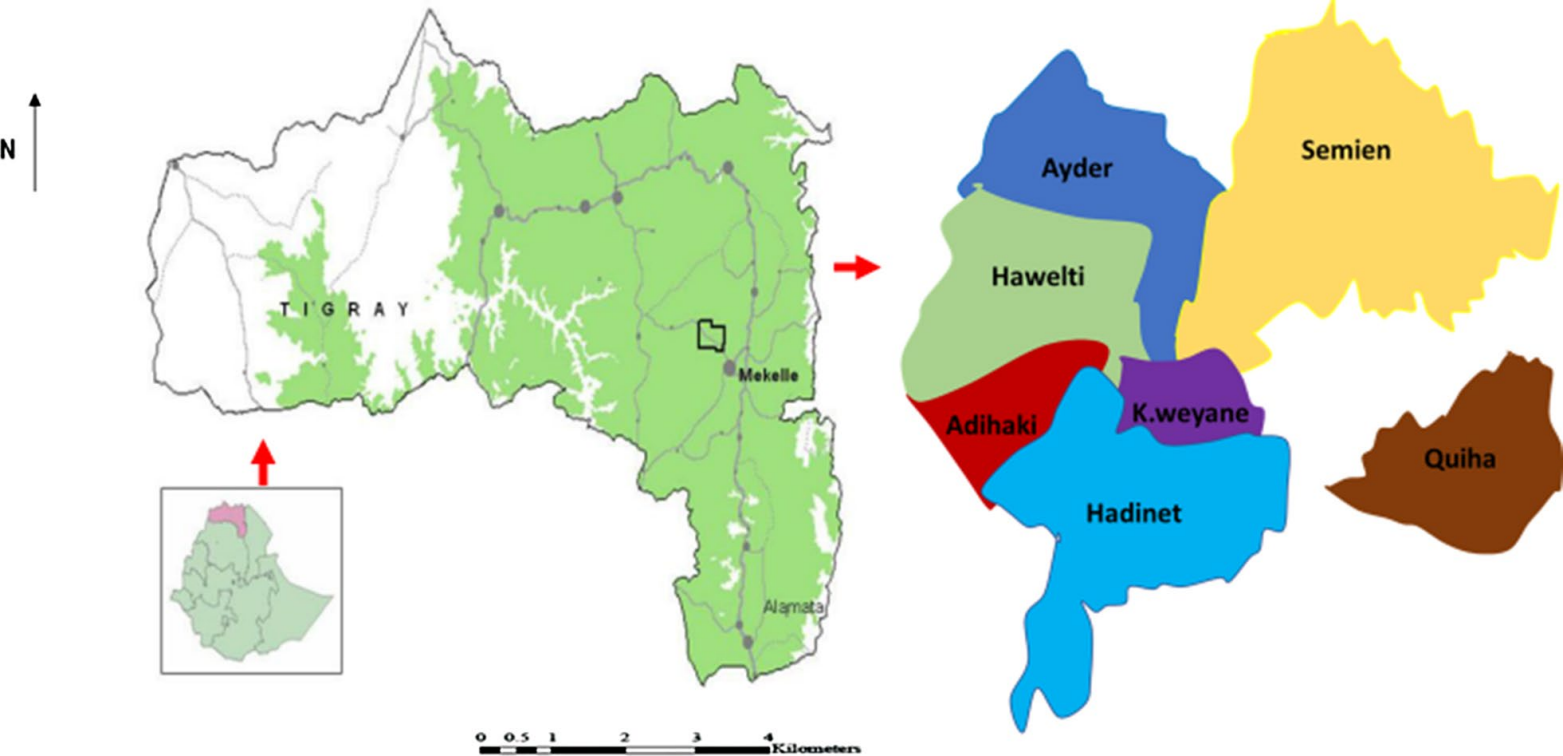
Geographic locations of the study area

The study was conducted in private dairy farms. As shown in Fig. 1, modified from the local organizing committee of the International Congress Water 2011 [15], the study area is located in Northern part of Ethiopia at a distance of 780 Km from Addis Ababa. It is located between 33°24′13″ to 13°36′52″ north latitude and 39°25′30″ to 39°38′39″ east longitude. It has an altitudinal range of 2150–2270 m above sea level. The average daily temperature is 11–24.1 °C.

#### Sampling strategies

A simple random sampling method was used to identify the number of dairy farms from the study area. All dairy farms having a size of greater or equal to five animals were registered and accordingly, 81 dairy farms were listed. From this list, randomly, a total of 30 dairy farms (18 private, 11 cooperative and 1 institution owned) were selected for the study. For the follow up studies, throughout the study period (6 months), all selected farms were regularly attended and diagnosed by veterinarians and farm managers and a total of 68 animals were randomly selected and from these, new cases were identified to classify the type of health problems. Neonatal calves were also included in the study subjects to assess any health problems. Any abnormalities and diseases conditions during clinical surveillance and follow-up period was recorded. We considered two types of breed: local breed which includes the boran and zebu cattle and exotic or Holstein–Friesian breeds.

#### Clinical diagnosis

Individual cases were screened for the presence of disease with the help of veterinarians in the clinic and basic laboratory methods such as floatation and sedimentation were used for characterizing parasites following the procedure given by Hansen and Perry [14] and the remaining diseases types were identified based on the clinical judgement by the veterinarian. After confirmation, clinical cases were administered with recommended therapeutics for treatment.

#### Data management and analysis

All data collected from the study were analyzed using statistical software for social sciences version 20 (SPSS 20) and Chi square statistical method was used to analyze the data. Descriptive statistical tools were used to show the incidence rate of different diseases. P-values less than 0.05 was considered as measure of statistical significance.

### Results

In this study, a total of 475 dairy cows were examined for major dairy health problems using a cross-sectional survey and 68 dairy cows using regular follow up studies and the result showed that a total of 205 (43.00%) of animals were affected by at least one or more major dairy health problems (infectious, reproductive and injury related problem). During follow up studies, 20 (29%) were examined for at least one of the dairy health problems. As shown in Table 1, the study of cross sectional survey revealed major infectious diseases such as respiratory problem (7.80%), mastitis (5.13%), actinomycosis (5.12%) and dystocia (4.42%), and retained fetal membrane (3.61%). Biting fly (9.51%), tick infestation (2.91%), endoparasite (3.81%) are the main parasitic problems encountered during the study period. Whereas, lameness (2.94%), and skin problem (1.70%) were the major animal injuries recorded. The assessment of major reproductive health problems revealed an incidence of 3.57, 4.43, 2.54, 1.70, 0.62, 0.83% for retained fetal membrane, dystocia, vaginal and uterine prolapse, abortion, repeat breeding, and ketosis respectively. The regular follow up study (Table 1) showed an incidence as follows: respiratory problem (2.92%), mastitis (2.93%), actinomycosis (1.50%), retained fetal membrane (5.93%), dystocia (2.91%), biting fly (1.53%), tick infestation (5.92%), endoparasite (2.94%), lameness (2.94%), skin problem (1.53%), vaginal and uterine prolapse (0%), abortion (0%), repeat breading (0%), and ketosis (1.5%). An incidence of parasitic infections, and other problems was also recorded during the follow up study and revealed as: nematodes (2.92%), biting fly (1.53%), tick infestation (5.90%), fasciolosis (1.53%), confirmed by laboratory, skin problem (1.52%), hernia (1.52%), and lameness (2.91%). Of the total animals examined, 26.00% of local breeds had infection and 21% injuries and related problems. Whereas, 22.23% of the exotic breeds had infection and 59.00% injuries. Animals under intensive and semi intensive management system were found to have 22.00, 10.00% of infectious problems respectively and, 21.61, and 14.42% injury related problems respectively (Table 2). The incidence of reproductive, parasitic, and injurious problem in relation to breeds was significant (P < 0.05). However, incidence of infectious diseases was not significant with breed of animal (P > 0.05). Management system resulted significant difference (P < 0.05) in the occurrence of infectious, parasitic, and injurious problem which was high at semi-intensive than intensive management system. The incidence of reproductive problem was not significant (P > 0.05) with respect to management system.

**Table 1:** Incidence of major dairy animal diseases based on cross-sectional survey and follow up studies

Type of clinical case	Frequency	Incidence (%)
Cross-sectional survey		
Mastitis	24	5.1
Actinomycosis	24	5.1
Calf diarrhea	10	2.1
Respiratory problem	37	7.8
Eye infection	10	2.1
Retained fetal membrane	17	3.6
Dystocia	21	4.4
Vaginal and uterine prolapse	12	2.5
Abortion	8	1.7
Repeat breading	3	0.6
Ketosis	4	0.8
Endoparasite	18	3.8
Biting fly	45	9.5
Tick infestation	14	3
Fasciolosis	6	1.3
Skin problem	8	1.7
Hernia	2	0.4
Lameness	13	3
Follow up study		
Mastitis	2	3
Actinomycosis	1	1.5
Calf diarrhea	2	3
Respiratory problem	2	3
Eye infection	0	0
Retained fetal membrane	4	6
Dystocia	2	3
Vaginal and uterine prolapse	0	0
Abortion	0	0
Repeat breading	0	0
Ketosis	1	1.5
Endoparasite	2	3
Biting fly	1	1.5
Tick infestation	4	6
Fasciolosis	1	1.5
Skin problem	1	1.5
Hernia	1	1.5
Lameness	2	3

**Table 2:** Infectious, RP, parasitic and injury cases in dairy cows: RP, reproductive problem and Mg, management

Risk factor breed and Mg	No. of cow examined	No. with infectious (%)	X^2^ (P-value)	No. with RP (%)	X^2^ (P-value)
Local	85	26 (30.5)	2.75 (0.07)	21 (24.7)	7.97 (0.05)
Cross	458	102 (22.2)		59 (12.8)	
Total	543	128 (23.5)		80 (14.7)	
Intensive	58	22 (37.9)	35.55 (0.00)	10 (17)	32 (0.56)
Semi intensive	485	105 (21.6)		70 (14.4)	
Total	543	127 (23.3)		80 (14.7)	

Risk factors breed and Mg	No of cows examined	No with parasite (%)	X^2^ (P-value)	No with injurious (%)	X^2^ (P-value)
Local breed	85	11 (12.9)	14.07 (0.00)	32 (37.6)	24.14 (0.00)
Cross breed	458	20 (4.4)		69 (15)	
Total	543	31 (5.7)		101 (18.6)	
Intensive	58	29 (50)	35.55 (0.00)	9 (15.5)	16.39 (0.00)
Semi intensive management	485	81 (16.7)		17 (3.5)	
Total	543	110 (20.2)		26 (4.78)	

### Discussion

In the present study, the incidence rate is different depending on the nature of the disease whether it is infectious, non-infectious diseases or reproduction related problem. The productive problems were the most frequently encountered cases during the study. This is due to the poor management of dairy animals during milking and poor husbandry practices. For example, all farms use the manual or non-machinery milking system, and this enhances the degree of contamination and the chance of transmitting pathogens among dairy animals. Regular sanitation and applying a pre-milking teat dip or spray for the prevention of infection is not a common practice in the farms. Dystocia was frequently observed during this study period and there are many factors associated with it. Such as the maternal factors (the size of the birth canal) and fatal factors (Orientation and over sizing of the foetus). The problem associated with dystocia was found to be associated with cause of retained fatal membrane in certain clinical cases. But many hormonal factors might also contribute for the problem. Similar studies also showed that retained fetal membrane was one of the problems of dairy cows with an incidence of 5–8% [7]. Furthermore, the present study showed a lower incidence of abortion comparing to previous studies [8, 9]. This is because dairy cows in the present study area gets a regular treatment with antibiotics and we suggest the therapeutics helped in controlling the major abortion causing diseases such as brucellosis. Moreover, physical injury was also identified as a cause of early abortion in dairy cows. Infectious diseases were also occurred frequently at an incidence of ranging from 5.00 to 10.71% which is in agreement with other reports conducted in the southern region of Ethiopia [10]. One of the justification for increasing the incidence of infectious diseases in the area is due to the non-selective administration of therapeutic drugs which has an impact on the efficacy of treatment outcomes. The incidence of respiratory problems such as pneumonia, bronchitis, and sinusitis were found lower than the previous studies [11]. The housing quality which is spacious and well aerated might contribute for the findings of low incidence in our study. Moreover, incidence of actinomycosis in the present study revealed 6.52% which is also in agreement the previous studies [12]. Parasitic diseases were observed as common health problem of livestock in the area. Tick infestations, and endoparasites were found frequently to cause loss of body condition. The high occurrence of parasitic disease in the study area could be due to low deworming practice as we have obtained this information from the farm owners. Whereas, incidence of tick infestation recorded in this study agrees with another report which showed an incidence of 8.8% [13]. Finally, injurious problem were important health challenges of cattle which is due to uncontrolled hygiene, production and management system and in this study, different types of physical injuries such as lameness, bruising, and injuries as a result of physical damage during restraining for milking and medication was assessed by the veterinarians and the result showed its significant impact on the wellbeing of dairy animals.

### Conclusion

In conclusion, this study identified a wide range of infectious and noninfectious dairy animal diseases which are the main threat for the dairy health sector in the region. Based on this finding, detail studies on the dynamics of the diseases should be studied, and prompt control measure should be in action.

### Limitations of the study

The lack of adequate laboratory facilities to diagnose all kind of animal disease was one of the limitations in this study. As a result, we are unable to make sure about the specific pathogen as there could be a high chance of mixed infections and this might affect the true map of the disease distribution in the area.

## Abbreviations

CSA: Central Statistical Authority
DVM: Doctor of Veterinary Medicine

## References

[1] Central Statistical Agency (CSA) (2009). The 2002/2003 Ethiopian agricultural enumeration (ESAE), executive summary, May 2009.

[2] Tambi EN, Maing OW, Mukhebi AW, Randolph TF. Economic impact assessment of render pest control in Africa. In: With the assistance of PARC epidemiological unit and PARC communication unit report paper. 1999.

[3] Shitaye JE, Tsegaye W, Paulik I (2007). Bovine tuberculosis infection in animal and human population in Ethiopia: a review. Vet Med.

[4] Abebe W, Esayas G (2001). Servey on ovine and caprine gastrointestinal Helminthosis in Eastern part of Ethiopia during dry season of the year. Revue Vet Med..

[5] Michael T (1986). Veterinary epidemiology.

[6] Tedla M, Gebreselassie M (2018). Estimating the proportion of clinically diagnosed infectious and non-infectious animal diseases in Ganta Afeshum Woreda, Eastern Tigray Zone, Ethiopia. BMC Res Notes..

[7] Correa MT, Curtis CR, Erb HN, Scarlett JM, Smith RD (1990). An ecological analysis of risk factors for postpartum disorders of Holstein–Friesian cows from thirty-two New York farms. J Dairy Sci.

[8] Berihu H, Abebaw G (2009). Major reproductive health problems of dairy cattle in and around Bako West Ethiopia. Ethiop J Anim Prod..

[9] Tekelye B, Kasali OB, Gashaw T (1992). Reproductive problems in indigenous cattle of the ministry of agricultural farm in central Ethiopia. Trop Agric (Trinidad).

[10] Tedla M, Degefa K (2017). Bacteriological study of calf colisepticemia in alage dairy farm, southern Ethiopia. BMC Res Notes..

[11] Steal SJ, Shapiro BI. Impacts of dairy input and output price policy on producer incentive in Ethiopia. In: Processing of the third national conference of the Ethiopian society of animal production (ESAP), April 27–29. Addis Ababa: Addis Ababa University, Ethiopia; 1995. p. 24–42.

[12] Tesfahiwot Z. Major health problem of livestock in the yarer water shed, Adaaliben worada. South Eastern Shoa. DVM, thesis FVM. Addis Ababa: AAU Debre Zeit, Ethiopia; 2004.

[13] Ameni G. Erko B. Bogale T. Preliminary study on major bovine trematode infections around kemisse northern Ethiopia and treatment trial with Praziquantel. In: Bull animal health production Africa. 2001. p. 62–7.

[14] Hansen J, Perry B (1994). The epidemiology, diagnosis and control of helminth parasites of ruminants. A hand book.

[15] International Congress. Water 2011 integrated water resources management in tropical and subtropical drylands, Mekelle, Ethiopia, 19–26 September 2011.

